# When modern diagnostics is challenged by a historical disease: A case report

**DOI:** 10.1097/MD.0000000000030586

**Published:** 2022-09-16

**Authors:** Marta Jankowska, Krystian Mross, Marcin Pałczyński, Karolina Machowska-Sempruch, Anna Bajer-Czajkowska, Miłosz Parczewski, Marta Masztalewicz

**Affiliations:** a Department of Neurology, Pomeranian Medical University, Szczecin, Poland; b Department of Infectious, Tropical Diseases and Acquired Immunodeficiency Pomeranian Medical University, Szczecin, Poland.

**Keywords:** case report, neuroimaging, neuroinfection, neurosyphilis, sexually transmitted diseases

## Abstract

Syphilis is a bacterial, systemic infectious disease caused by Treponema pallidum spirochetes, which spread rapidly through the body affecting various organs. The term neurosyphilis (NS) refers to a CNS infection that can occur at any stage of the disease. The lack of a gold standard for the diagnosis of NS greatly hinders diagnosis, which must be based mainly on clinical assessment.

**Patient concerns:**

A 58-year-old man reported dizziness and headache for a week and right-sided hearing impairment, with suspected transient cerebral ischemic attack. A month later he experience transient speech disturbance and suspected cerebral ischemic stroke.

**Diagnosis:**

MRI showed fresh ischemic lesions with a diameter up to 10 mm in the deep brain structures on the left side and foci of subacute ischemia also in the deep structures and the brain stem. Cerebrospinal fluid examination showed positive Pandy’s reaction, doubtful Noone-Apelt reaction, increased protein level and decreased glucose level. The reactive result of the USR test performed (VDRL) finally allowed the diagnosis of symptomatic CNS syphilis.

**Interventions:**

Empiric treatment for bacterial meningitis was administered. The patient was transferred to the Department of Infectious Diseases for further treatment.

**Outcomes:**

The diagnosis has been confirmed at the Department of Infectious Diseases after repeating CSF analysis including VDRL and FTA-ABS.

**Lesson:**

Symptoms of NS are nonspecific, hence the diagnostic process is not straightforward. Despite the availability of modern diagnostic techniques, establishing a final diagnosis was challenging, but the patient ultimately received appropriate treatment. It is important to remember that syphilis is not only a disease known from history lessons but is still present in modern times and its incidence is increasing.

## 1. Introduction

Syphilis is a bacterial systemic infectious disease caused by the spirochete Treponema pallidum, subspecies pallidum (T. pallidum). The discovery of penicillin, which replaced mercury and other substances used in the treatment of syphilis, made it possible to control the spreading of the disease.^[1]^ However, over the last decades, an increase in the incidence of syphilis has been noted.^[2]^ Statistics in Poland show that the incidence has risen from 3.3 cases per 100,000 population in 2009 to 6.0 cases per 100,000 population in 2019.^[3]^

Syphilis can be divided into primary, secondary latent (hidden), and tertiary (late) syphilitic stages based on clinical findings.^[4]^ T. pallidum is spreading rapidly through the organism after initial infection and can affect any organ, hence the disease can have multiple presentations. Neurosyphilis (NS) is a term referring to the infection of the central nervous system (CNS) that may occur at any time of infection.^[5]^ The incidence of NS is hard to estimate precisely since most data concerning the natural history of neurosyphilis predates the penicillin era. Some researchers report, that it develops in about 30% of people with untreated syphilis.^[6]^ In more recent studies the percentage of symptomatic NS varies between 3.5 to 13%.^[7]^

In modern times, neurosyphilis is most commonly seen in patients with HIV.^[8]^ However, the manifestations in HIV-infected and uninfected patients are similar.^[9]^

Some researchers called neurosyphilis “the great imitator” since its manifestation can mimic many different disorders.^[10]^ Symptoms of neurosyphilis can be divided into 2 groups: early and late. Early neurosyphilis usually occurs weeks to the first few years after initial infection and includes asymptomatic neurosyphilis, symptomatic (acute syphilitic) meningitis, gumma, and meningovasculitis. Whereas late neurosyphilis manifests in paretic neurosyphilis and tabes dorsalis.^[11]^

Despite many modern diagnostic techniques and medical technologies available any gold standard for the diagnosis of neurosyphilis has not been created. The diagnosis of neurosyphilis is quite challenging and must rely mainly on clinical judgement as there is no single laboratory test that is both highly sensitive and specific for NS.^[12]^

**Figure 1.**
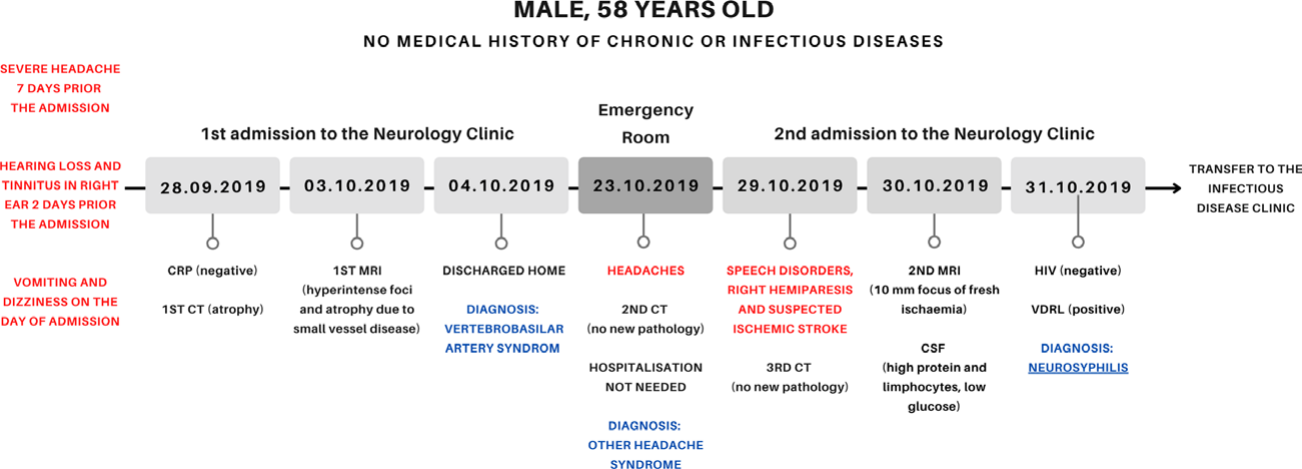
Timeline.

In this article, we presented an unusual case of NS in a patient without HIV infection or any other kind of immunodeficiency and diagnostic challenges (Fig. 1). We would like to emphasize, that syphilis is still present in modern times and neurosyphilis can develop even in patients without risky sexual behavior in anamnesis.

## 2. Case presentation

In September 2019, a 58-year-old male presented to the Emergency Department with the chief complaint of a headache persisting for 7 days. On the day of the admission dizziness, vomiting and balance disturbance appeared early in the morning. The patient also complained of hearing loss and tinnitus in the right ear for the last 2 days. He was a nonsmoker, without any history of drug or alcohol abuse, with no medical history of chronic or infectious diseases. He had undergone surgery due to duodenal ulcers in the 90s. Laboratory tests detected only anemia, however, CRP and other parameters were within normal range. Neurological examination revealed left-beating nystagmus (grade 3.) and wide-based, unsteady gait. The patient underwent an otolaryngology examination and hearing test, which confirmed hearing impairment in the right ear but did not reveal the cause of other symptoms. The patient was treated with betahistine, steroids, potassium chloride and omeprazole.

**Figure 2.**
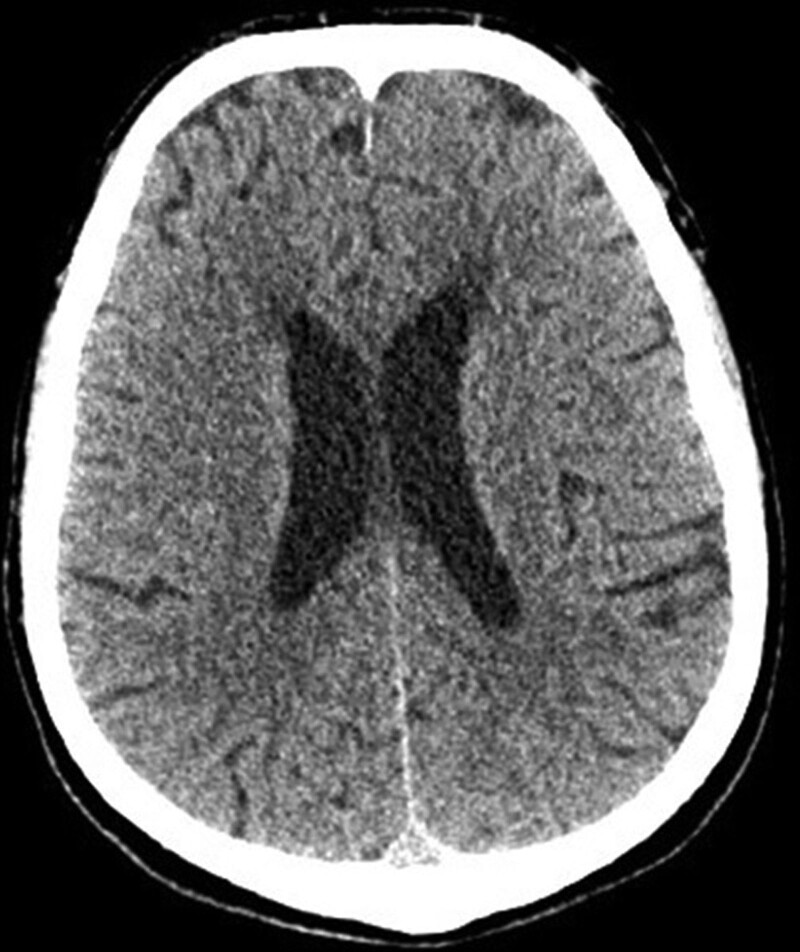
Head CT scan of the patient at first hospitalization.

A computed tomography (CT) of the head showed cortical-subcortical atrophy (Fig. 2), but no other pathology.

Contrast-enhanced magnetic resonance imaging (CEMRI) of the brain revealed scattered hyperintense foci on T2 images, FLAIR sequence in the right cerebral hemisphere periventricularly and deep structures and in the brainstem with features of water diffusion restriction in DWI images, which indicated fresh micro-infarcts. Despite them, in the white matter of both cerebral hemispheres and periventricularly hyperintense foci on T2 images and FLAIR sequence were detected, interpreted as degenerative changes on the background of small vessel disease. Also, minor cortical-subcortical atrophy was presented.

Doppler ultrasonography was also performed, revealing diffuse heterogeneous atherosclerotic plaques with foci of calcification (haemodynamically insignificant) in the carotid arteries, hypoplastic right vertebral artery with a diameter of 1.4 mm and high resistive blood flow spectrum with reduced velocity. In comparison, the left vertebral artery was 3.6 mm in diameter with a patent, normal blood flow spectrum.

Vertebrobasilar artery syndrome was diagnosed. After a six-day hospitalization, the patient’s condition improved and then he was discharged home with a recommendation for a follow-up visit to the Neurology and Otolaryngology Ambulatory Care.

In October 2019, the patient reached our Emergency Department with a headache for the last few days. He was without any abnormalities in the neurological examination. Computed tomography of the head was performed, but no new pathology was found in comparison to the CT scan from the previous hospitalization. Intracranial hemorrhage was also excluded. The patient was discharged home.

**Figure 3.**
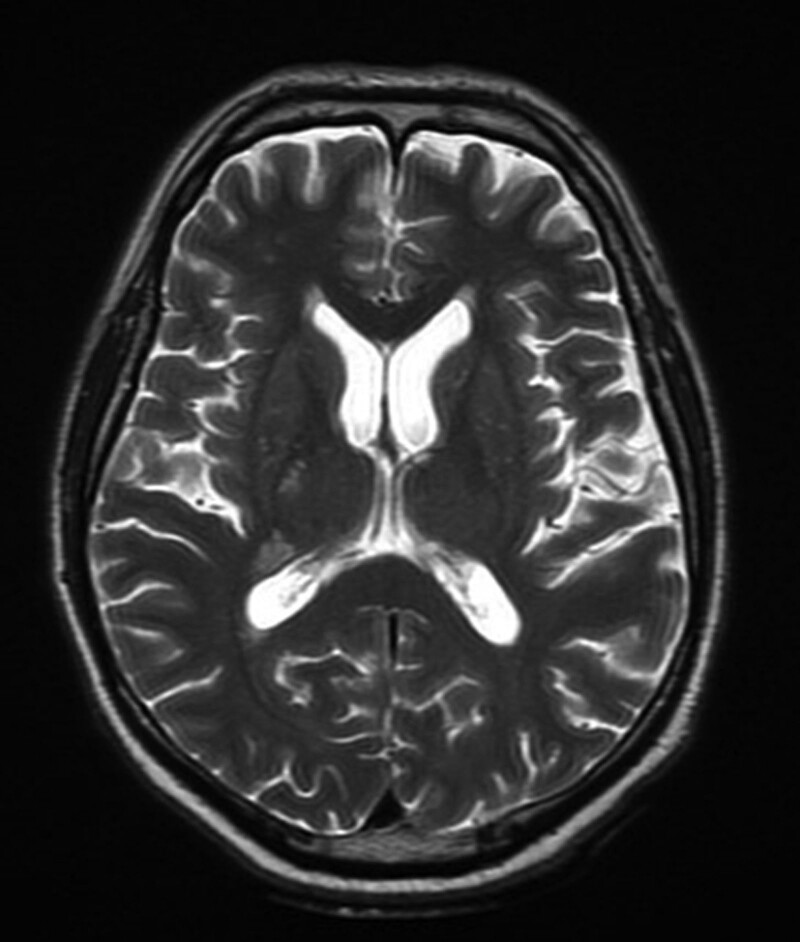
MRI scan of the brain with hyperintense foci of subacute ischemia in the deep structures of the right hemisphere (FLAIR sequence).

Six days later the patient was admitted to our hospital with suspicion of stroke. A few hours prior to admission, during the planned neurological consultation patient had experienced transient speech disturbance. While being examined by the emergency medical team, the patient was conscious, with psychomotor slowness, circulatory and respiratory efficient. In physical neurological examination hyperreflexia and spasticity in left limbs have been observed. Meningeal signs were absent. Laboratory tests revealed a borderline concentration of leukocytes (9.14 thousand/ul), slightly reduced Na (132 mmol/l) and insignificantly raised CRP (5.43 mg/l). A subsequent CT scan of the brain showed no changes compared to previous examinations, while head MRI showed fresh ischemic lesions (hyperintense on T2, FLAIR and DWI images) with a diameter up to 10 mm in the deep brain structures (thalamus and internal capsule) on the left side. Hyperintense foci of subacute ischemia in the deep structures of the right hemisphere and the brain stem on T2 images, FLAIR and DWI images with slight contrast enhancement have been also described (lesions in previous head MRI described as acute) (Fig. 3).

Apart from the changes mentioned above, the MRI image was comparable to the MRI performed during the previous hospitalization. The whole clinical presentation and neuroimaging have given rise to suspicion of a chronic inflammatory process, possibly a vasculitis or neuroinfection. Therefore a decision was made to perform a lumbar puncture. The analysis of the cerebrospinal fluid (CSF) revealed deviations typical for lymphocytic meningitis (Table 1). During the diagnostic process, the patient received empirical antibiotic therapy as with meningitis. Testing for human immunodeficiency virus (HIV) was found to be negative, but blood Venereal Disease Research Laboratory (VDRL) tested positive. The laboratory test results indicating Treponema pallidum infection and the results of the cerebrospinal fluid test supported the diagnosis of meningovascular syphilis. With such suspicion, the patient was referred to the Department of Infectious Diseases, where the diagnosis has been confirmed after repeating CSF analysis including VDRL and FTA-ABS (Table 2). Proper treatment has been administered and the patient remained under the observation in Infectious Diseases Ambulatory Care.

**Table 1:** Characteristics of the cerebrospinal fluid (CSF) test findings in the Neurology Clinic.

General characteristics			
Colour CSF	Colourless		
Appearance CSF	Clear		
Clot	None		
	Test result	Unit	Normal range
Protein	114,20	mg/dl	15 – 45
Glucose	33,00	mg/dl	50 – 80
Chlorides	115,00	mmol/l	113 – 127
Cells			
Lymphocytes	69	/ul	0 – 5
Granulocytes	3	/ul	0 – 5
Borreliosis IgG	<0,2000	AU/ml	<4,5
Borreliosis IgM	0,68	AU/ml	< 2,5
Nonne-Apelt Test	weakly positive (+)		
Pandy Test	positive (+++)		

**Table 2:** Characteristics of the cerebrospinal fluid (CSF) test findings in the Infectious Diseases Clinic.

General characteristics			
Colour CSF	Colourless		
Appearance CSF	Clear		
Clot	None		
	Test result	Unit	Normal range
Protein	121,20	mg/dl	15–45
Glucose	52,00	mg/dl	40–70
Chlorides	118,00	mmol/l	115–130
WBC	71,00	/μl	0–5
FTA	1/320		
FTA-ABS	positive		
VDRL	1/8 (+++)		

## 3. Discussion

Available studies report that in HIV-negative patients male gender and age ≥ 45 years are both risk factors for neurosyphilis.^[13]^ The patient presented in this case report had both of those risk factors. However, no medical history of sexually transmitted diseases or risky sexual behaviors has not brought NS into consideration, which ultimately prolonged the diagnostic process and making the final diagnosis.

Nausea, vomiting, headache and vertigo are nonspecific early symptoms of meningovasculitis.^[14]^ Our patient experienced those symptoms, among others, during his first admission to the hospital.

Infectious cerebral vasculitis commonly presents as headaches, seizures, encephalopathy and even stroke. In 2012, Chinese researchers published a retrospective study conducted on a group of 149 neurosyphilis patients, of whom 14% developed ischemic stroke as a primary symptom.^[15]^ Some studies indicate, that the rate of misdiagnosis of neurosyphilitic ischemic stroke might be high since its clinical symptoms are similar to other diseases and show no differences in CT and MRI results.^[16]^ Hence, the suspicion of neurosyphilis in patients with TIA and ischemic stroke should be taken into consideration.

The plurality of symptoms and atypical manifestations of neurosyphilis causes many diagnostic challenges. Therefore, additional tests, including neuroimaging and laboratory tests, play a key role in the diagnostic process of the infection. Radiologic characteristics of neurosyphilis in MRI are not highly specific, however, MRI is very sensitive in detecting changes in the course of cerebral vasculitis, usually including small and medium vessels.^[17]^ Neuroimaging findings in neurosyphilis include white matter lesions or cortical and subcortical infarction.^[18]^ Other manifestations of meningovascular syphilis including hypointensity on T1, hyperintensity on T2, medium contrast enhancement, perilesional edema, meningeal enhancement/dural tail, arteritis, leptomeningeal granulomas (known as gummata), extraaxial enhancement, high signal changes in the bilateral medial temporal lobes including both hippocampi and amygdalas on T2 images and FLAIR and DWI sequence, ventricular dilatation were documented.^[17]^ Nevertheless, as much as two-thirds of patients with neurosyphilis were reported to have a normal MRI, or a nonspecific mild to moderate cerebral atrophy.^[19]^ Cerebral atrophy might be the result of chronic meningoencephalitis.^[14]^ In our patient, both cortical-subcortical atrophy in CT of the head and hyperintense foci on T2 images in MRI of the brain were revealed during the diagnostic process. Those findings are nonspecific, even though are commonly reported in patients with neurosyphilis.

According to the guidelines, no single test can be used to diagnose neurosyphilis in all instances. The diagnostic process should include a combination of CSF tests (CSF cell count or protein, and a CSF-VDRL) in the setting of reactive serologic test results and neurologic signs and symptoms.^[9]^ CSF abnormalities consistent with neurosyphilis have been variably defined. However, the most important CSF abnormalities in patients with neurosyphilis include elevated protein and white blood cell count (leucocytes).^[20]^ Moreover, diagnosis of neurosyphilis usually relies on reactive CSF-VDRL, which has high specificity but low sensitivity. Unfortunately, as seen in some studies, many cases of likely active neurosyphilis lack a reactive CSF-VDRL test. CSF-VDRL can be false-negative in up to 70% of cases, especially in late neurosyphilis.^[21]^ Another systematic review indicates, that positive CSF-VDRL may be the most specific test in diagnosing neurosyphilis, but using it as the sole criterion to define neurosyphilis may affect a study’s clinical applicability since 50% of persons treated for neurosyphilis in clinical practice have a negative CSF-VDRL.

Penicillin G remains the treatment of choice for syphilis.^[9]^ According to the CDC guidelines, the preferred regimen is 18 to 24 million units of aqueous crystalline penicillin G per day, administered as 3 to 4 million units IV every 4 hours or continuous infusion, for 10 to 14 days. Alternative regiments include procaine penicillin G plus probenecid.^[22]^

Other considerations in the management of patients with neurosyphilis should be taken, especially testing for HIV. Although neurosyphilis is most commonly diagnosed in HIV-positive patients, the patient in our case tested negative.

## 4. Conclusions

Neurosyphilis can be a potentially serious complication of syphilis that can occur at any stage of the T. pallidum infection and can imitate symptoms of many different diseases. Even though syphilis and its implications have been well known over the centuries, there is still a lack of a “golden standard” for the diagnosis of neurosyphilis. Neuroimaging can be useful in the diagnostic process, however, it cannot certainly confirm the etiology of the pathologic changes in the MRI or CT. Hence, CSF analysis remains the main tool in the diagnostic process and should be conducted in any atypical and problematic case. Penicillin is the most important drug used in the treatment of neurosyphilis.

## Author contributions

Marta Jankowska participated in the study concept and design, analysis and interpretation of data, and preparation of the manuscript. Krystian Mross participated in the acquisition of data, analysis and interpretation of data, and preparation of the manuscript. Marcin Pałczyński participated in the preparation of the manuscript. Karolina Machowska-Sempruch participated in the acquisition of data. Anna Bajer-Czajkowska participated in the acquisition of data.

Miłosz Parczewski participated in the acquisition of data. Marta Masztalewicz participated in the study concept and design, analysis and interpretation of data, and preparation of the manuscript.
